# The inflammation–immunosuppression loop as a driver of immune dysfunction and secondary infections in severe COVID-19

**DOI:** 10.3389/fimmu.2026.1934930

**Published:** 2026-08-27

**Authors:** Guochao Zhang, Yanjun Lai, Yingying Zhao

**Affiliations:** 1 Department of Clinical Laboratory, Ninth Hospital of Xi’an, Xi’an, Shaanxi, China; 2 Department of Pathology, Shanxi University of Medicine, Fenyang, Shanxi, China

**Keywords:** COVID-19, IL-6–JAK–STAT3 signaling, immune network remodeling, immune paralysis, myeloid-derived suppressor cells (MDSCs), SARS-CoV-2, secondary infections, TNF-α–NF-κB pathway

## Abstract

Severe COVID-19 is strongly associated with a high incidence of secondary bacterial, fungal, and viral infections, which substantially contribute to prolonged hospitalization and increased mortality. Although cytokine storm characterized by elevated levels of IL-6, TNF-α, and IL-1β has long dominated the understanding of the immunopathology of severe COVID-19, accumulating clinical and mechanistic evidence indicates that persistent immunosuppression and impaired antimicrobial immunity are equally critical determinants of disease outcomes. The coexistence of excessive inflammatory activation and profound immune paralysis within the same patient represents a major unresolved paradox in COVID-19 immunology. In this review, we systematically summarize recent advances in the mechanisms underlying SARS-CoV-2-induced immune remodeling and propose an integrated signaling network model that links inflammatory amplification with immunosuppressive reprogramming. We highlight the central roles of the GM-CSF, MAPK, TNF-α–NF-κB, and IL-6–JAK–STAT3 signaling networks in sustaining inflammatory activation, driving myeloid cell reprogramming, impairing antigen presentation, and promoting T-cell dysfunction and exhaustion. Furthermore, we propose an “inflammation–immunosuppression loop” model, in which persistent inflammatory signaling actively induces immunosuppressive states through STAT3-dependent transcriptional programs, while impaired antimicrobial immunity facilitates secondary infections. Infection-associated activation of pattern recognition receptors subsequently reinforces inflammatory signaling, establishing a self-amplifying positive feedback loop that accelerates disease progression. This model provides a mechanistic explanation for the paradoxical coexistence of hyperinflammation and immune paralysis in severe COVID-19 and redefines secondary infections as active drivers of disease deterioration rather than merely downstream complications. Finally, we evaluate current and emerging host-directed therapeutic strategies and propose that future immunotherapeutic approaches should shift from single cytokine blockade toward precision restoration of immune homeostasis guided by dynamic monitoring of immune network states.

## 1 Introduction

Coronavirus disease 2019 (COVID-19), caused by severe acute respiratory syndrome coronavirus 2 (SARS-CoV-2), is a highly contagious infectious disease that has imposed an unprecedented burden on global public health since its emergence in 2019. Although widespread vaccination and the implementation of antiviral therapies have substantially reduced the incidence of severe disease and mortality, severe COVID-19 remains a major global health challenge. Increasing clinical evidence indicates that secondary bacterial, fungal, and viral infections following SARS-CoV-2 infection have become major determinants of prolonged hospitalization, intensive care unit (ICU) admission, multiple organ dysfunction syndrome (MODS), and mortality and mortality (1–3). The incidence of these secondary infections increases dramatically with disease severity. Ventilator-associated bacterial pneumonia (VAP) affects approximately 15–50% of critically ill patients (4, 5); COVID-19-associated pulmonary aspergillosis (CAPA) occurs in 5–30% of ICU patients and is associated with attributable mortality exceeding 50% (6, 7); and reactivation of latent herpesviruses, including cytomegalovirus (CMV), Epstein–Barr virus (EBV), and herpes simplex virus (HSV), has been reported in 25–60% of critically ill patients (8–10).

Early investigations primarily focused on the cytokine storm hypothesis, in which markedly elevated levels of circulating IL-6, TNF-α, and IL-1β were considered central drivers of systemic inflammation and multi-organ injury (11–13). However, accumulating clinical observations have revealed a profound immunological paradox that cannot be fully explained by excessive inflammation alone. Despite persistent systemic inflammatory activation, patients with severe COVID-19 simultaneously exhibit profound lymphopenia, impaired antigen-presenting capacity of dendritic cells and monocytes, marked expansion of myeloid-derived suppressor cells (MDSCs), functional exhaustion of virus-specific T cells, and extensive reactivation of latent pathogens (14–16). The coexistence of sustained inflammatory activation and progressive immune suppression within the same host strongly suggests that the immunopathology of severe COVID-19 is not simply a consequence of uncontrolled inflammation, but rather represents a dual pathological process characterized by the simultaneous activation of inflammatory and immunosuppressive programs. Systems immunology approaches have provided critical insights that cytokines and intracellular signaling pathways do not function as independent entities but instead operate cooperatively within highly integrated regulatory networks. Among these networks, the IL-6–JAK–STAT3 signaling axis has emerged as a central molecular hub connecting upstream inflammatory pathways, including GM-CSF, MAPK, and TNF-α–NF-κB signaling, with downstream immunosuppressive programs such as MDSC expansion and T-cell exhaustion (17–21). Persistent activation of this signaling network may therefore provide a mechanistic link between inflammatory amplification, immune dysfunction, and susceptibility to secondary infections.

In this review, we propose that severe COVID-19 should be conceptually redefined as a disease of immune network remodeling rather than merely a disorder of excessive cytokine production. We establish a unified molecular framework centered on the inflammation–immunosuppression loop to elucidate the complex interplay between immune dysregulation and secondary infection susceptibility in critically ill patients. This perspective provides a new mechanistic paradigm for understanding disease progression and highlights the need for immune network-guided therapeutic strategies beyond conventional cytokine-targeted interventions.

## 2 Dynamic evolution of host immune responses during SARS-CoV-2 infection: from antiviral defense to immune paralysis

The host immune response to SARS-CoV-2 infection is not a simple linear process but rather a dynamic continuum driven by the coordinated interplay of antiviral defense, inflammatory amplification, and immune functional remodeling (**Table 1**). During the early phase of infection, the host initiates antiviral defense programs through innate immune mechanisms. Following viral entry into respiratory epithelial cells, SARS-CoV-2-derived RNA is recognized by multiple pattern recognition receptors (PRRs), including Toll-like receptors (TLR3, TLR7/8, and TLR9) and cytosolic RNA sensors such as retinoic acid-inducible gene I (RIG-I) and melanoma differentiation-associated protein 5 (MDA5). Activation of these sensing pathways induces the production of type I and type III interferons (IFNs), which restrict viral replication and promote the establishment of antiviral immunity (22–26). However, SARS-CoV-2 has evolved sophisticated immune evasion strategies that impair early antiviral responses. Multiple viral nonstructural and accessory proteins, including NSP3, NSP6, NSP12, ORF3a, ORF6, and ORF7b, interfere with PRR recognition, suppress IFN signaling cascades, and inhibit the expression of downstream interferon-stimulated genes. Consequently, delayed or insufficient IFN responses permit sustained viral replication and increase the risk of tissue injury (27–32). As viral replication persists and tissue damage accumulates, an excessive inflammatory phase emerges. During this stage, activated macrophages, monocytes, neutrophils, and T cells collectively generate a highly pro-inflammatory cytokine milieu characterized by elevated levels of IL-6, TNF-α, IL-1β, GM-CSF, CXCL8, and CCL2 (33–38). Persistent inflammatory stimulation promotes further immune cell recruitment and activation, leading to alveolar epithelial injury, endothelial dysfunction, and systemic inflammatory responses involving multiple organs (39). Importantly, the immunopathological progression of severe COVID-19 does not represent a state of continuous immune activation. Instead, prolonged inflammatory stimulation gradually drives a transition toward a state in which inflammation and immune suppression coexist, commonly referred to as immune paralysis. Notably, this transition should not be interpreted as a strictly sequential temporal event; rather, it represents a dynamic immune remodeling process with substantial interindividual variability in onset and severity. Longitudinal studies have demonstrated that immunosuppressive features may emerge during the early stages of disease and can coexist with ongoing hyperinflammatory responses (40, 41).

**Table 1:** Dynamic evolution of host immune responses during SARS-CoV-2 infection.

Immune phase	Key mechanisms	Major outcomes
Antiviral defense	PRR-mediated recognition induces IFN responses to restrict viral replication	Early viral control
Viral immune evasion	Viral proteins suppress IFN signaling and antiviral pathways	Persistent viral replication and tissue injury
Inflammatory activation	Immune cell activation promotes cytokine production (IL-6, TNF-α, GM-CSF)	Inflammation, lung injury, and organ dysfunction
Immune remodeling/paralysis	Immune exhaustion, impaired antigen presentation, and immunosuppressive programs	Reduced pathogen clearance and secondary infections

The transition from inflammation toward immune paralysis is regulated by a combination of host-related and environmental factors, resulting in substantial heterogeneity among patients. Age, underlying comorbidities, vaccination status, and therapeutic interventions all influence the trajectory of immune responses during COVID-19. Elderly individuals, characterized by immunosenescence and inflammaging, exhibit both elevated baseline inflammatory activity and impaired immune competence, including reduced T-cell reserves, compromised antigen-presenting capacity, and limited immune recovery potential. Consequently, older patients are particularly susceptible to a pathological state characterized by simultaneous inflammatory activation and immune deficiency (42). Comorbid conditions such as diabetes, obesity, and cardiovascular diseases further accelerate disease progression through mechanisms involving metabolic inflammation, endothelial dysfunction, and impaired immune regulation (43, 44). In addition, vaccination status and therapeutic strategies substantially shape the immune trajectory of COVID-19. Vaccine-induced neutralizing antibodies and memory T-cell responses reduce viral replication and consequently limit excessive inflammatory amplification; however, their protective effects are influenced by age and immune status (45). Immunomodulatory therapies, including corticosteroids, IL-6 receptor blockers, and Janus kinase (JAK) inhibitors, can effectively attenuate inflammatory injury, but their clinical benefits depend critically on treatment timing and the underlying immune state of individual patients (46). Excessive or premature immune suppression may further compromise antiviral defense and increase susceptibility to opportunistic infections.

## 3 Integrated signaling networks driving inflammation and immunosuppression in severe COVID-19

**Figure 1:**
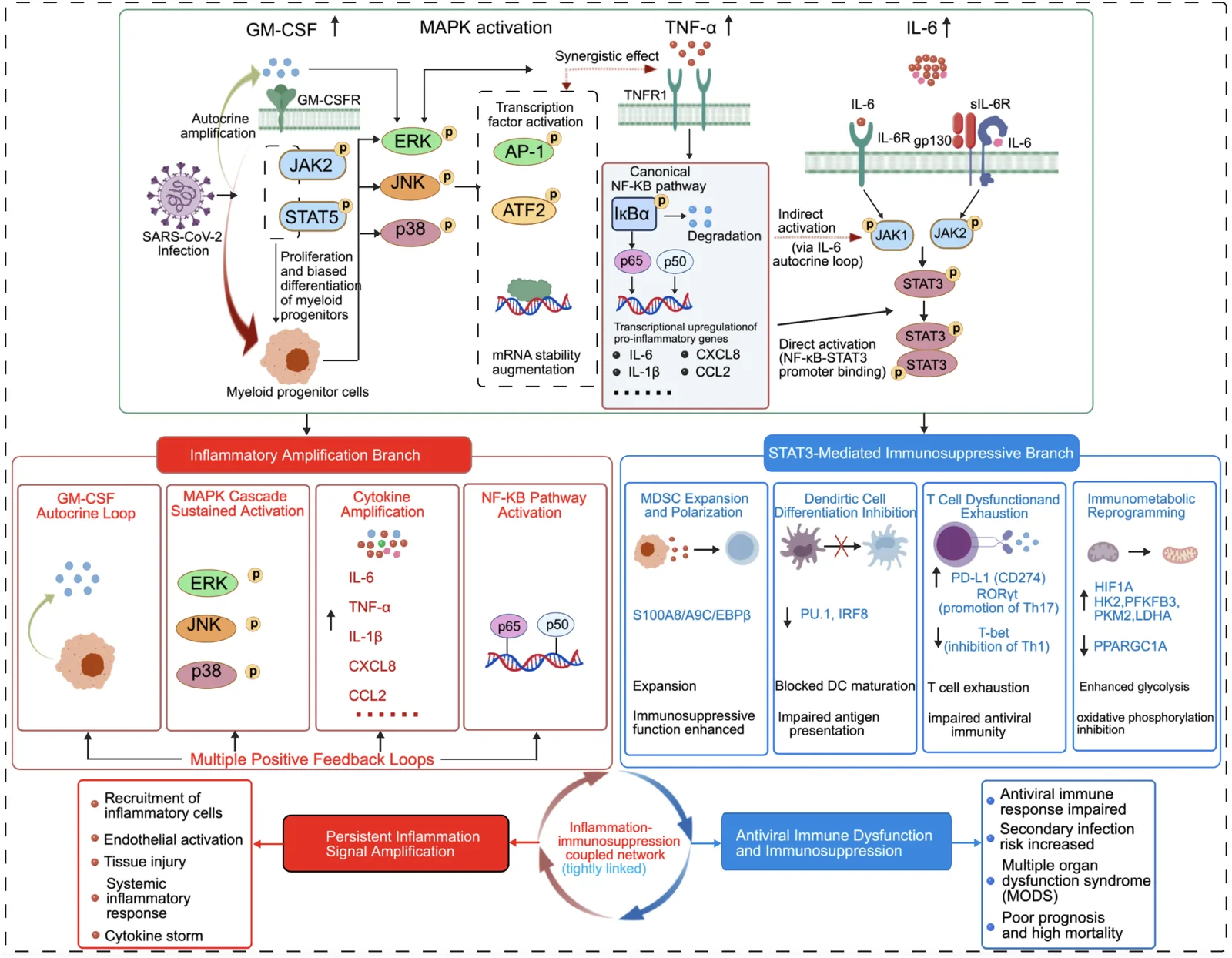
Schematic diagram of the inflammation–immunosuppression loop driving inflammatory amplification and immunosuppressive remodeling in severe COVID-19.

Severe COVID-19 is increasingly recognized not as a disease caused solely by excessive inflammatory cytokine production, but as a dynamic process of immune remodeling orchestrated by interconnected signaling networks (22, 47). Multi-omics analyses of peripheral blood from patients with severe COVID-19 have demonstrated extensive alterations in inflammatory pathways, myeloid cell programs, and immune regulatory networks, supporting the concept of systemic immune dysregulation rather than isolated cytokine overproduction (47). Increasing evidence suggests that GM-CSF, MAPK, TNF-α–NF-κB, and IL-6–JAK–STAT3 signaling pathways do not operate independently but constitute an integrated regulatory network coordinating inflammatory amplification and immune suppression (48, 49). Among these pathways, GM-CSF and IL-6 appear to represent major inflammatory regulators in severe COVID-19, whereas STAT3 has been proposed as a central molecular node linking acute inflammation with subsequent immune dysfunction (48, 50). However, the precise interactions among these pathways and their causal contribution to immune paralysis in COVID-19 remain incompletely defined (**Figure 1**).

### 3.1 Inflammatory amplification network: GM-CSF, MAPK, and TNF-α–NF-κB signaling

Clinical studies have consistently demonstrated elevated circulating GM-CSF concentrations in patients with severe COVID-19, which correlate with disease severity, inflammatory burden, and unfavorable clinical outcomes (51). These observations have stimulated therapeutic investigations targeting GM-CSF signaling as a potential host-directed intervention strategy (52). Mechanistically, pro-inflammatory cytokines including IL-1β, IL-6, and TNF-α can induce GM-CSF production, while GM-CSF further enhances myeloid progenitor expansion, differentiation, and inflammatory polarization through JAK2–STAT5 and MAPK signaling pathways (53, 54). Activated monocytes and macrophages exposed to GM-CSF exhibit increased expression of inflammatory mediators, including TNF-α, IL-1β, and IL-6, suggesting that GM-CSF may function as both a consequence and an amplifier of inflammatory activation.

SARS-CoV-2 infection also induces substantial activation of MAPK signaling, particularly the ERK, JNK, and p38 MAPK pathways (55, 56). Phosphoproteomic analyses in SARS-CoV-2-infected cellular models have directly demonstrated activation of MAPK-related signaling, including p38 MAPK activation (55). Activated MAPK pathways enhance inflammatory gene expression through transcriptional regulators such as AP-1 and ATF2 and through regulation of inflammatory mRNA stability, thereby promoting production of IL-6, TNF-α, CXCL8, and CCL20 (56, 57). In addition, p38β/MAPK11 signaling has been implicated in facilitating SARS-CoV-2 replication, indicating that MAPK activation may simultaneously regulate host inflammatory responses and viral replication processes (55, 57). Collectively, the GM-CSF–MAPK network represents an important component of the early inflammatory amplification program during severe SARS-CoV-2 infection.

The TNF-α–NF-κB axis further reinforces this inflammatory environment. SARS-CoV-2-induced NF-κB activation promotes transcription of TNF-α, IL-6, and other inflammatory mediators, establishing a classical positive-feedback inflammatory loop (58, 59). NF-κB activation has been directly observed in SARS-CoV-2-infected pulmonary epithelial models, supporting its involvement in virus-induced inflammatory responses (58). Importantly, accumulating evidence suggests that NF-κB signaling may also serve as a transition point between inflammation and immune dysfunction. By promoting IL-6 production, NF-κB enhances downstream JAK–STAT3 activation, while sustained inflammatory signaling may further reinforce STAT3-dependent transcriptional programs (48, 59). Thus, persistent NF-κB activation may contribute not only to inflammatory amplification but also to the establishment of an immunosuppressive state.

### 3.2 Immune suppressive transition: IL-6–JAK–STAT3 signaling and MDSC expansion

The IL-6–JAK–STAT3 pathway represents a critical molecular axis involved in the transition from inflammatory activation toward immune suppression. IL-6 activates JAK1/JAK2 kinases through both classical signaling via membrane-bound IL-6 receptors and trans-signaling mediated by soluble IL-6 receptor complexes, resulting in STAT3 phosphorylation at Tyr705, dimerization, and nuclear translocation (60, 61). Activated STAT3 regulates a broad range of genes involved in inflammation, immune regulation, and cellular metabolism (48, 50). Clinically, elevated IL-6 levels are strongly associated with respiratory failure, systemic inflammation, and disease progression in severe COVID-19, supporting the therapeutic relevance of IL-6–STAT3 signaling in this disease (35, 37). Importantly, STAT3 exhibits context-dependent functions during SARS-CoV-2 infection. While transient STAT3 activation may contribute to inflammatory defense responses, persistent STAT3 signaling has been associated with immune suppressive programs and impaired antimicrobial immunity (48, 50). One major consequence of sustained STAT3 activation is the expansion and functional polarization of MDSCs, a heterogeneous population of immature myeloid cells with potent immunoregulatory activity (62, 63).

Both monocytic MDSCs (M-MDSCs) and granulocytic MDSCs (G-MDSCs) accumulate in peripheral blood and respiratory compartments of patients with severe COVID-19, and their abundance correlates with disease severity, ICU admission, and mortality (16, 62, 64, 65). STAT3 signaling has been implicated in MDSC development through induction of transcriptional programs involving S100A8/S100A9 and C/EBPβ, while suppressing differentiation associated factors such as PU.1 and IRF8 (62, 64). Consistent with this model, S100A8-high and HLA-DR-low monocyte phenotypes, characteristic of immunosuppressive myeloid states, have been observed in severe COVID-19 patients (66, 67). These alterations are associated with impaired antigen presentation and features of immune paralysis. MDSCs suppress antiviral and antimicrobial immunity through multiple complementary mechanisms. Increased ARG1 activity results in local L-arginine depletion, reduced CD3ζ chain expression, and impaired T-cell proliferation and effector function (62). MDSC-derived iNOS-generated reactive nitrogen species can disrupt T-cell receptor signaling, whereas NADPH oxidase-derived ROS impair immune synapse formation and promote T-cell dysfunction (62, 64). Furthermore, MDSCs produce immunoregulatory cytokines such as IL-10 and TGF-β and express immune checkpoint molecules including PD-L1, thereby promoting regulatory T-cell expansion and T-cell exhaustion (63). Although these mechanisms are well established in cancer and chronic inflammatory diseases, their precise contribution to COVID-19 associated immune dysfunction requires further direct validation.

Beyond transcriptional regulation of immune responses, STAT3 signaling may contribute to immune dysfunction through metabolic remodeling. SARS-CoV-2 infection induces metabolic alterations characterized by increased glycolytic activity and reduced mitochondrial oxidative phosphorylation (OXPHOS), potentially mediated through HIF-1α-dependent pathways and PI3K–AKT–mTOR signaling (68, 69). Although enhanced glycolysis supports rapid immune activation, persistent metabolic reprogramming may result in mitochondrial dysfunction, reduced metabolic flexibility, and impaired long-term immune competence (70, 71). STAT3 has been implicated as an important regulator of this metabolic transition. Experimental studies indicate that STAT3 can enhance HIF1A transcription and increase expression of glycolytic enzymes, including HK2, PFKFB3, PKM2, and LDHA, while suppressing PPARGC1A (encoding PGC-1α), a key regulator of mitochondrial biogenesis and fatty acid oxidation (72, 73). These alterations may contribute to mitochondrial membrane potential loss, excessive ROS production, and disturbed mitochondrial dynamics (71, 74). In severe COVID-19, mitochondrial dysfunction in circulating immune cells has been demonstrated; however, the direct causal relationship between STAT3 activation and metabolic failure remains to be fully established. Metabolic competition between MDSCs and T cells may further reinforce immune suppression. MDSCs exhibit enhanced glycolytic activity and increased glucose uptake through GLUT upregulation, potentially reducing glucose availability within inflammatory niches (69, 70). Since activated T cells also depend on glycolysis to sustain proliferation and IFN-γ production, nutrient competition may contribute to impaired T-cell responses (68, 69). In addition, ARG1-mediated arginine depletion and IDO1-mediated tryptophan catabolism may promote regulatory T-cell differentiation through AhR activation, further stabilizing an immunosuppressive microenvironment (75).

## 4 Immune remodeling and secondary infection susceptibility in severe COVID-19

Secondary infections represent a major determinant of mortality and poor clinical outcomes in patients with severe COVID-19 (76, 77); however, their occurrence cannot be solely attributed to healthcare-associated factors such as mechanical ventilation, ICU exposure, or broad-spectrum antibiotic administration (4, 6, 78, 79). Increasing immunological evidence indicates that persistent immune remodeling induced by SARS-CoV-2 infection represents a critical intrinsic driver of secondary bacterial, fungal, and viral infections (22). This dysfunctional immune state is characterized by impaired pathogen recognition, defective antigen presentation, altered immune cell recruitment, and compromised effector functions, ultimately rendering patients vulnerable to persistent opportunistic infections (14, 16). Therefore, secondary infections should not be regarded merely as passive consequences of immune injury in severe COVID-19, but rather as active contributors that may further exacerbate immune dysfunction and disease progression (78, 80). Clinical disease kinetics provide important clues regarding the association between immune suppression and secondary infections. In most critically ill COVID-19 patients, secondary infections commonly occur during the second to third week after disease progression, a period that frequently coincides with the emergence of immunosuppressive features, including persistent lymphopenia, reduced monocyte HLA-DR expression, abnormal expansion of myeloid populations, and T-cell functional exhaustion (65, 67, 78, 79, 81–83). Longitudinal immune profiling studies have demonstrated that pronounced immunosuppressive signatures can be detected as early as 7–14 days after symptom onset (11, 47) in some patients, and the peak window of secondary infection susceptibility largely overlaps with this immune hyporesponsive phase. Although temporal association alone does not establish causality, the consistency between immune dynamics and infection timing strongly suggests that SARS-CoV-2-induced immune remodeling creates a permissive environment for opportunistic pathogen invasion (78, 79).

Immunophenotypic studies further support the concept that immune paralysis contributes to secondary infection susceptibility. Among various immune markers, reduced monocyte HLA-DR expression has been recognized as a hallmark of immune paralysis in COVID-19 (78, 84). Decreased HLA-DR levels reflect impaired antigen-presenting capacity of monocytes, resulting in insufficient T-cell activation and compromised pathogen clearance. Patients with persistently low HLA-DR expression exhibit increased susceptibility to secondary infections and are associated with higher mortality risk (78, 84). Dysfunction of adaptive immunity further amplifies infection susceptibility. Critically ill COVID-19 patients frequently exhibit sustained depletion of CD4^+^ and CD8^+^ T cells, T-cell exhaustion, and abnormal expression of immune checkpoint molecules (81, 85). In parallel, reduced B-cell numbers and impaired antibody production further weaken humoral immune protection against bacterial and fungal pathogens (41). The simultaneous impairment of innate and adaptive immunity ultimately establishes a state of profound immune paralysis, substantially increasing the likelihood of opportunistic infections. From a network perspective, SARS-CoV-2-induced immune remodeling and secondary infections may establish a bidirectional amplifying feed-forward loop. Persistent activation of the GM-CSF, MAPK, TNF-α–NF-κB, and IL-6–JAK–STAT3 signaling networks not only promotes inflammatory mediator production and tissue injury but also drives immunosuppressive reprogramming through STAT3-dependent MDSC expansion, impaired antigen presentation, and T-cell exhaustion (16, 48). Following invasion by secondary pathogens, pathogen-associated molecular patterns (PAMPs) can further stimulate pattern recognition pathways, including TLRs, C-type lectin receptors, and the cGAS–STING pathway (26, 86), thereby reactivating NF-κB, MAPK, and STAT3 signaling. This sustained activation reinforces the coexistence of inflammation and immunosuppression, generating a vicious cycle that accelerates disease deterioration. Therefore, secondary infections should not be considered merely as the endpoint of immune paralysis but rather as active participants that perpetuate immune dysregulation (**Table 2**).

**Table 2:** Immune remodeling and susceptibility to secondary infections in severe COVID-19.

Immune remodeling feature	Key mechanisms	Clinical consequences
Innate immune dysfunction	Impaired pathogen recognition, reduced monocyte HLA-DR expression, defective antigen presentation, and impaired phagocyte function	Decreased pathogen clearance and increased bacterial/fungal infection risk
Adaptive immune impairment	Persistent CD4^+^/CD8^+^ T-cell depletion, T-cell exhaustion, immune checkpoint activation, and reduced B-cell-mediated antibody responses	Increased susceptibility to opportunistic infections and viral reactivation
Inflammation–immunosuppression coexistence	Persistent activation of GM-CSF, MAPK, NF-κB, and IL-6–JAK–STAT3 networks promotes inflammation and STAT3-dependent MDSC expansion	Sustained immune dysregulation and disease progression
Secondary infection immune dysfunction feedback loop	Secondary pathogens activate PRRs (TLRs, CLRs, cGAS–STING), further enhancing NF-κB/MAPK/STAT3 signaling	Amplification of immune imbalance and clinical deterioration

Different types of secondary infections further illustrate the specific host defense defects caused by immune remodeling. Bacterial infections, particularly VAP, represent the most common secondary infections (4, 5) and frequently involve opportunistic pathogens such as Pseudomonas aeruginosa, Klebsiella pneumoniae, and Staphylococcus aureus (6, 7). Their development is closely associated with impaired alveolar macrophage phagocytosis, defective neutrophil antimicrobial activity, and disruption of airway epithelial barrier integrity (87). Fungal infections, especially CAPA, reflect a deeper impairment of antifungal immunity (6, 7). Suppression of STAT3-dependent Th17 responses results in reduced production of IL-17 and IL-22, weakening mucosal antifungal defenses. Meanwhile, impaired neutrophil recruitment and oxidative killing capacity limit the clearance of Aspergillus hyphae, and corticosteroid therapy may further extend this window of immune vulnerability (88). In addition, reactivation of latent viruses, including CMV, EBV, and HSV, is frequently observed in critically ill patients (8, 10). Such viral reactivation is closely associated with exhaustion of virus-specific CD8^+^ T cells and impaired natural killer (NK) cell cytotoxic activity (9). However, it is important to acknowledge that current evidence supporting a causal relationship between immune remodeling and secondary infections remains largely derived from clinical association studies. Although secondary infections occur more frequently in patients with profound immune dysfunction and are associated with unfavorable outcomes, ICU-related factors, including mechanical ventilation, vascular catheters, urinary devices, prolonged hospitalization, and antimicrobial exposure, may still act as potential confounders. Therefore, future studies integrating longitudinal immune monitoring, single-cell multi-omics approaches, and interventional trials are required to determine whether specific immune signatures can predict secondary infection risk and whether immune restoration strategies can improve clinical outcomes.

## 5 Therapeutic implications of immune network remodeling: toward precision host-directed therapy in severe COVID-19

The immune network remodeling model introduces new requirements for clinical therapeutic strategies. Rather than applying uniform treatment regimens, therapeutic interventions should be individually tailored according to the dynamic position of each patient along the inflammation–immunosuppression continuum (**Table 3**). During the hyperinflammatory phase, anti-inflammatory therapy remains a central component of treatment. Dexamethasone, through inhibition of NF-κB activation and suppression of inflammatory mediator production, has been demonstrated in the RECOVERY trial to significantly reduce mortality among hospitalized COVID-19 patients requiring respiratory support (89). The IL-6 receptor inhibitor tocilizumab and the JAK1/JAK2 inhibitor baricitinib have also demonstrated clinical benefits in patients with high inflammatory burden (90, 91), such as those with markedly elevated C-reactive protein (CRP >150 mg/L) or substantially increased D-dimer levels. These benefits include reduced risk of disease progression and accelerated clinical recovery. However, the timing of anti-inflammatory intervention is critical, as premature or excessive immune suppression may impair host antiviral defense mechanisms and increase susceptibility to secondary infections (35).

**Table 3:** Therapeutic strategies targeting immune network remodeling in severe COVID-19.

Immune state	Therapeutic strategy	Representative interventions	Potential benefits and considerations
Hyperinflammatory phase	Suppression of excessive inflammation	Dexamethasone, tocilizumab, baricitinib	Reduce inflammatory injury and disease progression; inappropriate timing may impair antiviral defense
Immunosuppressive phase	Immune restoration	PD-1/PD-L1 blockade, IFN-β, recombinant GM-CSF	Restore T-cell function, antiviral immunity, and myeloid cell function; clinical efficacy and safety require validation
Persistent immune dysfunction	Targeting immune network regulators and metabolism	STAT3 inhibitors, metabolic modulation strategies	Potentially reduce MDSC expansion and reverse immune metabolic reprogramming; risk of impaired host defense; clinical efficacy and safety require validation
Precision host-directed therapy	Sequential and individualized intervention	Anti-inflammatory control → immune restoration → metabolic regulation	Shift from pathogen-centered therapy toward restoration of immune homeostasis

For patients who have transitioned into an immunosuppressive state, immune restoration strategies are currently under active investigation, although no approach has yet been established as routine clinical practice. Immune checkpoint blockade targeting the PD-1/PD-L1 pathway, such as nivolumab, may potentially reverse T-cell exhaustion and restore virus-specific T-cell function (85), providing a therapeutic opportunity for persistent COVID-19 manifestations and prolonged SARS-CoV-2 infection. Recombinant interferon-β (IFN-β) administered during the early phase of disease may restore defective type I interferon responses (31, 92); however, its efficacy appears limited when administered after the peak inflammatory phase. Recombinant GM-CSF (sargramostim) theoretically has the potential to promote alveolar macrophage differentiation and functional recovery (52, 93), but its clinical application requires extremely careful timing to avoid exacerbating pre-existing inflammatory responses. Targeting STAT3 signaling represents a more integrated therapeutic strategy. Small-molecule STAT3 inhibitors may not only directly suppress MDSC expansion and immunosuppressive activity but also reverse STAT3 driven immune metabolic reprogramming and impaired dendritic cell differentiation (49, 50). Combination therapeutic strategies, such as sequential administration of JAK inhibitors followed by immune checkpoint blockade, or transition from adequate anti-inflammatory control toward immune restoration therapy, have been proposed as potentially more effective approaches than single-agent treatment (94). However, this concept remains hypothetical and requires clinical validation, as individual therapies are unlikely to simultaneously address the intertwined pathological processes of excessive inflammation and immune suppression. Importantly, all immune restoration strategies remain investigational in the context of COVID-19, and each approach carries potential risks. Immune checkpoint blockade may trigger excessive inflammatory toxicity; recombinant GM-CSF may aggravate pulmonary inflammation; and systemic STAT3 inhibition may compromise antifungal and antiviral immune defenses (50).

A major obstacle to implementing precision immunotherapy is the lack of clinically validated and reliable biomarkers capable of dynamically defining patient immune states in real time (47). Several promising candidate biomarkers include: (1) Monocyte HLA-DR expression, which directly reflects antigen-presenting capacity, with markedly reduced expression indicating an immunosuppressive phenotype (78); (2) Peripheral blood MDSC frequency, which reflects the degree of immunosuppressive myeloid expansion (16, 65); (3) T-cell PD-1 expression, which indicates the severity of T-cell exhaustion (8); (4) Serum lactate levels and arginine/tryptophan ratios, which reflect the extent of immune metabolic remodeling (70, 71). Integrating these biomarkers into bedside clinical decision-making algorithms may enable truly stage-specific immunotherapy. However, the optimal biomarker thresholds and therapeutic algorithms remain to be validated in prospective clinical studies. A sequential therapeutic strategy may therefore provide a rational framework for precision host-directed therapy in severe COVID-19: anti-inflammatory interventions during the hyperinflammatory phase, immune restoration approaches during the immunosuppressive phase, and metabolic modulation during persistent immune dysfunction. This individualized, immune-state-guided approach represents a potential paradigm shift from conventional pathogen-centered treatment toward dynamic restoration of immune homeostasis in severe COVID-19.

## 6 Discussion and future perspectives

The evidence synthesized in this review supports a paradigm shift in understanding the immunopathology of severe COVID-19. Rather than being merely a disorder caused by excessive cytokine production, severe COVID-19 should be recognized as a dynamic immune network remodeling syndrome (12, 13), in which the GM-CSF, MAPK, NF-κB, and JAK–STAT3 signaling networks constitute a central molecular framework integrating inflammatory amplification with immunosuppressive reprogramming. The conceptual model of the “inflammation–immunosuppression loop” provides a unified mechanistic explanation for the long-standing clinical paradox of simultaneous hyperinflammation and immune paralysis in critically ill patients (95). Within this framework, excessive inflammation and immune suppression are not independent pathological processes but are mechanistically coupled events. Persistent STAT3 activation converts inflammatory signals into immunosuppressive programs, including MDSC expansion, dendritic cell suppression, T-cell exhaustion, and metabolic reprogramming (16, 48). Meanwhile, impaired antimicrobial immunity facilitates secondary infections, which subsequently reactivate inflammatory pathways through persistent pattern recognition receptor (PRR) stimulation, thereby closing a self-amplifying pathogenic feedback loop (21, 26).

A key translational implication of this framework is that secondary infections should be considered an integrated component of disease progression rather than merely complications of severe COVID-19 (87). Prophylactic antimicrobial strategies and stringent infection-control measures may not only reduce acute mortality in critically ill patients but also interrupt the feed-forward cycle that perpetuates and deepens immune dysfunction. Notably, similar patterns of persistent STAT3 activation and MDSC expansion have been extensively reported in severe influenza, respiratory syncytial virus (RSV) infection (3, 96), and bacterial sepsis (95), suggesting that the “inflammation–immunosuppression loop”may represent an evolutionarily conserved pathogenic mechanism triggered by diverse infectious agents. Following the COVID-19 pandemic, increasing recognition of persistent immune abnormalities in post-acute sequelae of SARS-CoV-2 infection (PASC, commonly referred to as long COVID) has further highlighted the potential long-term consequences of immune network remodeling (97, 98). Persistent MDSC expansion, T-cell exhaustion, and metabolic dysregulation may continue for weeks or even months after viral clearance, suggesting that immune remodeling may contribute substantially to post-acute COVID-19 complications (99).

Several critical questions remain to be systematically addressed. First, the temporal dynamics and heterogeneity of immune network remodeling across different patient populations, including individuals with distinct ages, comorbidities, and genetic backgrounds, require investigation through large-scale, multicenter longitudinal immune profiling studies (41). Second, the proposed causal topology of the GM-CSF, MAPK, NF-κB, and JAK–STAT3 signaling network requires rigorous validation using conditional knockout animal models, lineage-tracing approaches, and systematic pharmacological perturbation studies. Third, reliable biomarker combinations capable of accurately distinguishing hyperinflammatory and immunosuppressive immune states remain insufficiently validated in clinical cohorts. Fourth, therapeutic interventions targeting immune network remodeling must carefully balance pathogen-specific immune requirements. For example, excessive inhibition of STAT3 signaling may increase susceptibility to fungal infections by impairing protective Th17 responses (100). Fifth, whether secondary infections causally aggravate immune remodeling or merely represent a consequence of immune dysfunction remains to be determined through prospective clinical cohorts adjusted for ICU-related confounding factors (83) and mechanistic interventional animal models.

Beyond molecular mechanisms, this framework has direct implications for treatment timing and long-term disease management. The transition from antiviral immunity toward maladaptive inflammation generally begins within the first week of infection; however, the precise timing and trajectory vary substantially among individuals. Therefore, fixed therapeutic windows should be regarded only as approximations rather than universal rules (101, 102). Early antiviral interventions, such as remdesivir, may limit viral replication and downstream inflammatory amplification (103, 104). However, once immune remodeling has been established, antiviral therapy alone is unlikely to reverse immune suppression and may require combination with immune-modulatory approaches. Increasing evidence indicates that some recovered individuals continue to exhibit immune abnormalities and increased susceptibility to infections even after viral clearance (97, 98), suggesting that immune network remodeling may have persistent clinical consequences. Furthermore, the continuous emergence of SARS-CoV-2 variants, including variants with enhanced immune escape capacity (105), further emphasizes the importance of understanding host immune regulatory mechanisms for the development of broadly protective therapeutic strategies. A major unresolved challenge remains clinical validation: immune-state-guided therapeutic approaches have not yet been sufficiently tested. Large prospective cohorts integrating longitudinal immune profiling with clinical parameters are required to translate these concepts into practical bedside decision-making tools.

From a broader systems biology perspective, immune network remodeling is not unique to COVID-19. Similar imbalances between inflammation and immune suppression occur in sepsis and chronic viral infections (79, 95) suggesting that shared mechanisms may facilitate cross-disease therapeutic development (106). Artificial intelligence and machine learning approaches applied to longitudinal immune profiling may eventually enable individualized prediction of immune trajectories and therapeutic responses; however, these approaches remain at an early research stage. Ultimately, the development of animal models that accurately recapitulate human immune remodeling dynamics, together with multicenter prospective immune monitoring studies, will be essential to validate this conceptual framework and transform it into clinically applicable decision-support strategies for precision immunotherapy.
